# Cortical Sensitivity to Guitar Note Patterns: EEG Entrainment to Repetition and Key

**DOI:** 10.3389/fnhum.2017.00090

**Published:** 2017-03-01

**Authors:** David A. Bridwell, Emily Leslie, Dakarai Q. McCoy, Sergey M. Plis, Vince D. Calhoun

**Affiliations:** ^1^The Mind Research NetworkAlbuquerque, NM, USA; ^2^Department of Electrical and Computer Engineering, University of New MexicoAlbuquerque, NM, USA; ^3^The MARC Program, University of New MexicoAlbuquerque, NM, USA

**Keywords:** mobile EEG, music, oddball, guitar, SSAEP, frequency tagging

## Abstract

Music is ubiquitous throughout recent human culture, and many individual's have an innate ability to appreciate and understand music. Our appreciation of music likely emerges from the brain's ability to process a series of repeated complex acoustic patterns. In order to understand these processes further, cortical responses were measured to a series of guitar notes presented with a musical pattern or without a pattern. ERP responses to individual notes were measured using a 24 electrode Bluetooth mobile EEG system (Smarting mBrainTrain) while 13 healthy non-musicians listened to structured (i.e., within musical keys and with repetition) or random sequences of guitar notes for 10 min each. We demonstrate an increased amplitude to the ERP that appears ~200 ms to notes presented within the musical sequence. This amplitude difference between random notes and patterned notes likely reflects individual's cortical sensitivity to guitar note patterns. These amplitudes were compared to ERP responses to a rare note embedded within a stream of frequent notes to determine whether the sensitivity to complex musical structure overlaps with the sensitivity to simple irregularities reflected in traditional auditory oddball experiments. Response amplitudes to the negative peak at ~175 ms are statistically correlated with the mismatch negativity (MMN) response measured to a rare note presented among a series of frequent notes (i.e., in a traditional oddball sequence), but responses to the subsequent positive peak at ~200 do not show a statistical relationship with the P300 response. Thus, the sensitivity to musical structure identified to 4 Hz note patterns appears somewhat distinct from the sensitivity to statistical regularities reflected in the traditional “auditory oddball” sequence. Overall, we suggest that this is a promising approach to examine individual's sensitivity to complex acoustic patterns, which may overlap with higher level cognitive processes, including language.

## Introduction

Music is composed of a series of repeated, complex acoustic patterns. Thus, our appreciation of music emerges from our ability to identify and represent complex streams of sounds. Traditional western music is composed of 12 notes with a fixed frequency interval between them, and a subset of seven notes comprise a key or diatonic scale (Krumhansl, 1990, 2000). Children and adults without musical training are sensitive to musical beat and scales, suggesting that individuals are predisposed to detect these musical features (Trehub et al., 1999; Brattico et al., 2006; Cirelli et al., 2016). Thus, the sensitivity to these features may be related to healthy development, and may overlap with the development of cognitive systems that support additional complex processes such as language and memory (Feld and Fox, 1994; Patel, 2011; Peretz et al., 2015).

An individual's cortical responses to music may be examined by measuring event-related potentials (ERP's) to rare sounds (e.g., notes) that differ from the musical context of surrounding sounds (e.g., by being out of key; Brattico et al., 2006; Miranda and Ullman, 2007). Using this “musical auditory oddball” approach, previous studies have demonstrated ERP response differences for changes in pitch (Brattico et al., 2001), timbre (Christmann et al., 2014), chord quality (Koelsch et al., 1999; Brattico et al., 2009; Tervaniemi et al., 2011; Virtala et al., 2014), harmony (Leino et al., 2007), or combinations of these features (Vuust et al., 2011, 2016). In general, these ERP response differences are reflected in a more negative ERP peak at ~100–250 ms (Koelsch, 2009; Yu et al., 2015), and an increase in the positive ERP peak that appears ~300–600 ms after the deviant stimulus (Besson and Macar, 1987; Nan et al., 2006).

Since ERP's are traditionally measured to discrete stimuli, the series of notes or chords are often presented one at a time, with intervals between them typically longer than 500 ms (for exceptions see Yabe et al., 1997; Wang et al., 2008; Loizides et al., 2015). This ≤ 2 Hz presentation rate ensures that traditional ERP peaks [e.g., the so-called “mismatch negativity” (MMN), “early right anterior negativity” (ERAN), and “P300”] to a given stimulus will not overlap with ERP's that emerge to subsequent stimuli (Luck, 2004). Unfortunately, the longer interval between successive notes or chords limits the musical qualities of the stimulus. Thus, there is motivation for measuring these responses using a faster presentation frequency, ensuring that the stimuli better align with the complexity, tempo, and perceptual qualities of traditional music (Tervaniemi and Brattico, 2004; Large, 2008).

Faster presentation rates influence both the neural and perceptual responses to auditory stimuli. With respect to EEG oscillations, qualitative differences in phase alignment appear when presentation frequencies exceed 1 Hz (Doelling and Poeppel, 2015). The faster presentation rate facilitates the entrainment of EEG oscillations to periodic stimuli, i.e., as demonstrated in frequency-tagging or steady-state evoked potential (SSEP) studies (Nozaradan et al., 2011, 2012, 2015; Thut et al., 2011; Bridwell and Srinivasan, 2012; Bridwell et al., 2013; Roth et al., 2013; de Graaf et al., 2013; Keitel et al., 2014). These “steady-state” cortical oscillations are related to comprehension and attention to music (Meltzer et al., 2015) and the ability to perceptually group stimuli presented close together in time (Carlyon, 2004; Van Zuijen et al., 2004).

Perceptual grouping may facilitate the ability to perceive the pattern within the auditory inputs and the ability to detect complex musical relationships (Nozaradan et al., 2011). For example, tempo sensitivity is optimal (i.e., follows Weber's Law) for tempo's from 1 to 5 Hz (Drake and Botte, 1993), individuals are sensitive to musical pulses up to 10 Hz (Repp, 2003), and maximum EEG synchronization appears between guitar duos playing to a metronome between 4 and 7 Hz (Lindenberger et al., 2009). Faster presentation rates also ensure that the stimuli overlap with temporal segmentation processes necessary for language, since speech prosody changes occur between 0 and 4 Hz and speech syllable boundaries occur between 4 and 8 Hz (Poeppel, 2003; Koelsch et al., 2005).

The present approach bridges the gap between traditional ERP designs and the steady-state design. Guitar notes were presented at 4 Hz with a random sequence or a patterned sequence within a blocked design. Amplitude reductions to the stimuli presented in musical patterns (compared to stimuli in random sequences), likely reflects individual's “cortical sensitivity” to musical patterns, since it indicates that ERP amplitudes are modulated by musical features, and greater processing resources are required when notes are presented without those features (i.e., randomly). Relationships between ERP amplitudes to guitar note patterns and ERP amplitudes within the traditional auditory oddball sequence were also examined. These findings demonstrate whether the cortical sensitivity to these patterns are distinct from the cortical sensitivity to statistical regularities reflected in the traditional “auditory oddball” sequence.

## Materials and methods

### Participants

Thirteen individuals (9 males; 1 left handed) between the ages 23 and 36 participated in a single experimental session. Each individual had normal audition and had no family history of mental illness. All participants were approved for EEG recordings by the University of New Mexico Institutional Review Board (HRRC/MCIRB), and provided informed written consent prior to the session at the Mind Research Network.

### Experimental design and stimuli

The session was comprised of five 5 min blocks, and individuals were instructed to listen to the sounds in each block. Within each block, guitar notes were presented through speakers at 4 Hz (i.e., note onsets are 250 ms apart). Blocks 1 and 2 consisted of a musical sequence of guitar notes, blocks 3 and 4 consisted of a random sequence of guitar notes, and block 5 consisted of a single guitar note presented frequently (90% of the time) and a second guitar note presented infrequently (10% of the time; see Supplementary Audio files).

**Figure 1:**
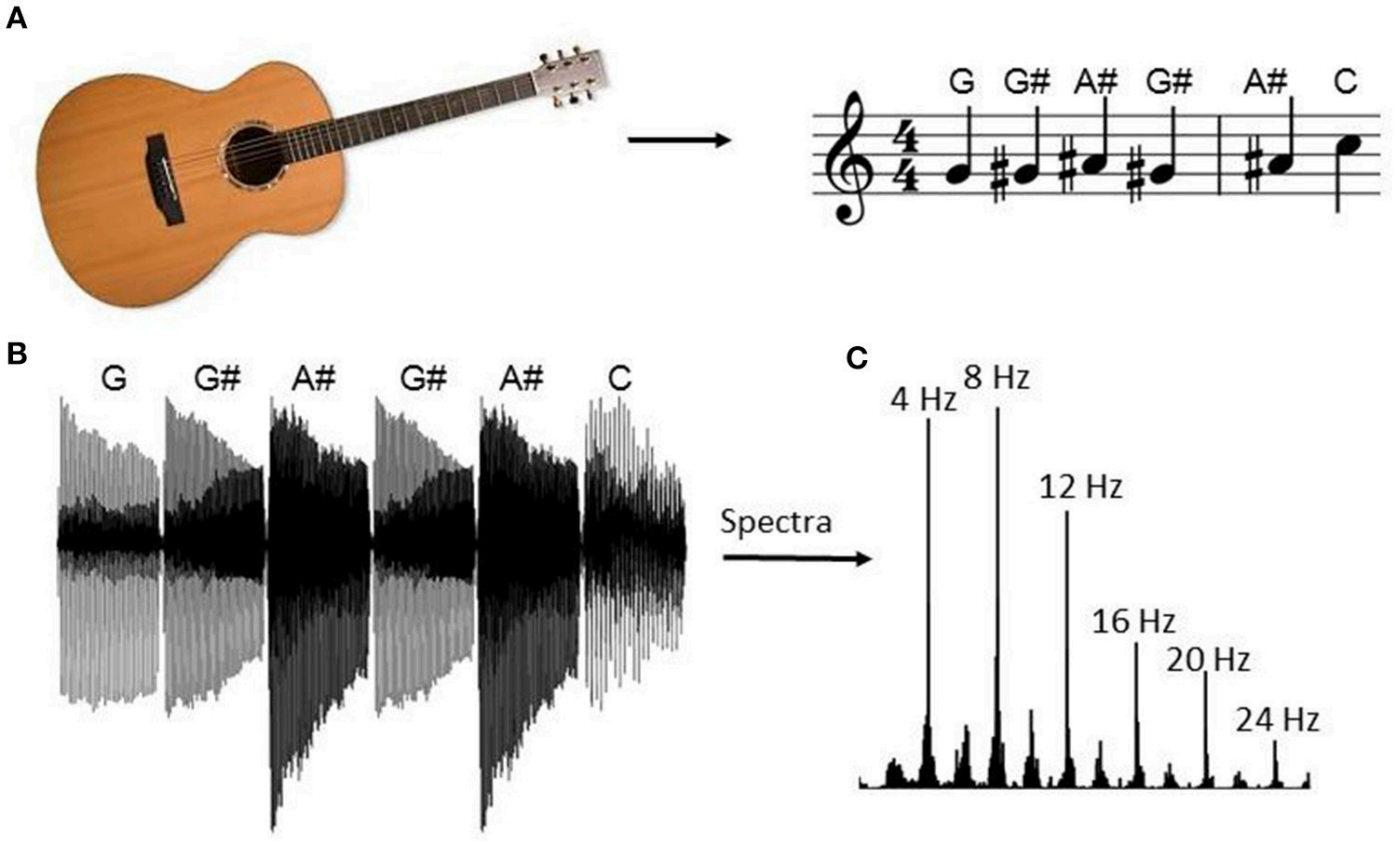
**Guitar note sequence**. During the blocks with musical structure (i.e., blocks 1 and 2) the experiment began by playing patterns drawn from the guitar scales with repetition. The sequence began by presenting notes within G# major drawn from the E major scale shape, as indicated in **(A)**. After the last note of the scale pattern, the sequence began again using the second scale pattern (D shape), followed by the third (C shape), and the fourth (A shape) pattern (not depicted). At the end of the fourth pattern the sequence repeated except each scale increased by one semitone (i.e., the pattern repeated in the key of A). The stimulus vector for the first six guitar notes is indicated in **(B)** and the spectral content of the entire sequence is indicated in **(C)**. The peak at 4 Hz corresponds to the note repetition frequency and the subsequent peaks correspond to its harmonics.

Within blocks 1 and 2, notes were presented within four scales with a repeating pattern. The block began by playing notes G-G#-A#-G#-A#-C-A#-C-C# etc. from the E shape guitar scale within the key of G# major (Figure 1). After reaching the end of the first scale, the same sequence was repeated using the second scale shape (D shape), then the third (C shape), and the fourth (A shape). The pattern was then repeated from the first shape after increasing the key by one semitone. The sequence of stimuli was identical within blocks 1 and 2.

The sequence within blocks 1 and 2 was randomized and presented within blocks 3 and 4. Thus, the stimuli are physically identical across the “pattern” and “random” conditions, ensuring that differences in ERP responses between these conditions are directly related to differences in sensitivity to the musical sequence (i.e., the order in which the notes appeared). The same sequence of random stimuli was presented across blocks 3 and 4 and across all subjects.

In the fifth block, ERP's were collected within an oddball sequence where the G note on the thickest string is presented 90% of the time (i.e., the “standard” stimulus) and the F note on the thinnest string is presented 10% of the time (i.e., the “oddball” stimulus). The trial sequence was random and identical across all subjects. Notes were presented at 4 Hz, consistent with blocks 1–4.

The order of the blocks was counterbalanced across participants by randomly selecting a sequence of stimuli from a row within a Latin square. The Latin square consisted of a 5 × 5 matrix with elements 1, 2, …5 such that no element repeated within a given row or column. Subjects were instructed to minimize movements and fixate on a central fixation dot for the duration of the experiment. The mean was removed from each audio stimulus, and amplitudes were normalized to 40% of the maximum. Each note took up the entire 250 ms interval except the first and last 10 ms were ramped on and off to avoid clipping (see Figure 1B).

### Software

We used the SMARTING Streamer (the software interface for mBrainTrain's Smarting EEG amplifier) to collect data. The amplifier is connected to the computer with Bluetooth manager BlueSoleil. Experimental stimuli are presented through MATLAB using the sound.m function. UDP triggers are sent from MATLAB prior to each stimulus and the Bluetooth EEG was synchronized to each trigger using the Lab Streaming Layer (LSL) (http://github.com/sccn/labstreaminglayer).

### EEG acquisition and preprocessing

EEG data was collected using a 24-channel SMARTING amp (mBrainTrain http://www.mbraintrain.com; sample rate = 500 Hz) and an EasyCap EEG cap. EEG activity was recorded using sintered Ag–AgCl electrodes placed according to the 10–20 International System, with CPz and Pz added [the common mode sense (CMS) and driven right leg (DRL) electrodes were located on FCz and Fpz, respectively]. Electrode impedances were kept below 20 kΩ.

EEG preprocessing was conducted in Matlab (http://www.mathworks.com) using custom functions, built-in functions, and the EEGLAB toolbox (http://sccn.ucsd.edu/eeglab). The EEG data was linearly detrended, forward, and backward filtered with a Butterworth filter (bandpass: 0.01–50 Hz), referenced to channel CZ for bad channel identification, and re-referenced to the average of the mastoids. Bad channels were identified based on the data distribution and variance of channels, as implemented in EEGLAB's pop rejchan.m function (Delorme and Makeig, 2004) and the FASTER toolbox (Nolan et al., 2010), and spherically interpolated. On average, 2.62 channels were removed (std = 5.06). Blink artifacts were attenuated by conducting a temporal ICA decomposition on the individual recordings (extended Infomax algorithm in EEGLAB; Bell and Sejnowski, 1995; Lee et al., 1999). Artifactual components were identified by visual inspection of the component time-course, topographic distribution, and frequency spectrum and removed from the back reconstructed time-course (Jung et al., 2000). On average, 5.77 components were removed (std = 2.89).

### ERP amplitudes

EEG signals were segmented within the interval −250 to 500 ms surrounding each stimulus. Artifactual epochs were identified using the automatic artifact epoch detection algorithm in EEGLAB (function: pop_autoreg.m), and the single trial peak amplitudes within these epochs were excluded in the subsequent analysis. Epochs were also removed if any Bluetooth packets were lost within the interval. On average, 11.49% of epochs were removed from blocks 1 to 4 (max: 31.83%; min: 03.10%) per subject, and 7.28% of epochs were removed from block 5 (max: 20.08; min: 01.83). Individual amplitudes were calculated by identifying the onset and offset times of each peak within the group-averaged data, identifying the peak within that region within each individual subject and averaging amplitudes surrounding the full width half maximum (fwhm).

### Self-reports

Individuals filled out a form designed to assess their musical experience and preference after completing the experiment. For the first item, individuals were asked to rate their familiarity with musical scales between 1 and 7 (in intervals of 1), with 1 indicating “not at all familiar,” 4 indicating “somewhat familiar,” and 7 indicating that they were “very familiar.” Next, they were asked to rate (from 1 to 7) the degree in which they liked music, with 1 indicating “no,” 4 indicating “indifferent,” and 7 indicating “I love it.” Next, they reported if they could identify the beat when listening to music by circling one of the following options: never, almost never, sometimes, almost always, and always. Finally, they were asked if they played an instrument, and if so, how many hours they have played in total.

### Statistical analysis

Statistical tests were conducted to examine differences in individual ERP amplitudes between the music and random conditions (4 *T*-tests for four peaks) and between ERP's to the rare and frequent note (2 *T*-tests for two peaks). All tests were conducted on electrode Fz since it contained the largest peak response averaged across random and patterned notes, and averaged across rare and frequent nodes (Averaging across conditions ensures that the electrode choice is unbiased with respect to differences between conditions). We then determined whether individual's sensitivity to music is related to their sensitivity to rare stimuli by correlating ERP amplitude differences between music and random conditions with the ERP amplitude to rare stimuli (2 Pearson correlation tests). Amplitude differences between the music and random conditions were linearly correlated with individual's self- reported familiarity with musical scales (2 Pearson correlation tests). These statistical tests are reported as “significant” if they pass Holm–Bonferroni correction for the 10 planned comparisons (alpha = 0.05) (i.e., the 10 uncorrected *p*-values are ordered and the lowest *p*-value is significant if it is below 0.05/10 = 0.005, the second lowest *p*-value is significant if it is below 0.05/9 = 0.0055, and so on; Holm, 1979).

## Results

### ERP differences to musical and random sequences

**Figure 2:**
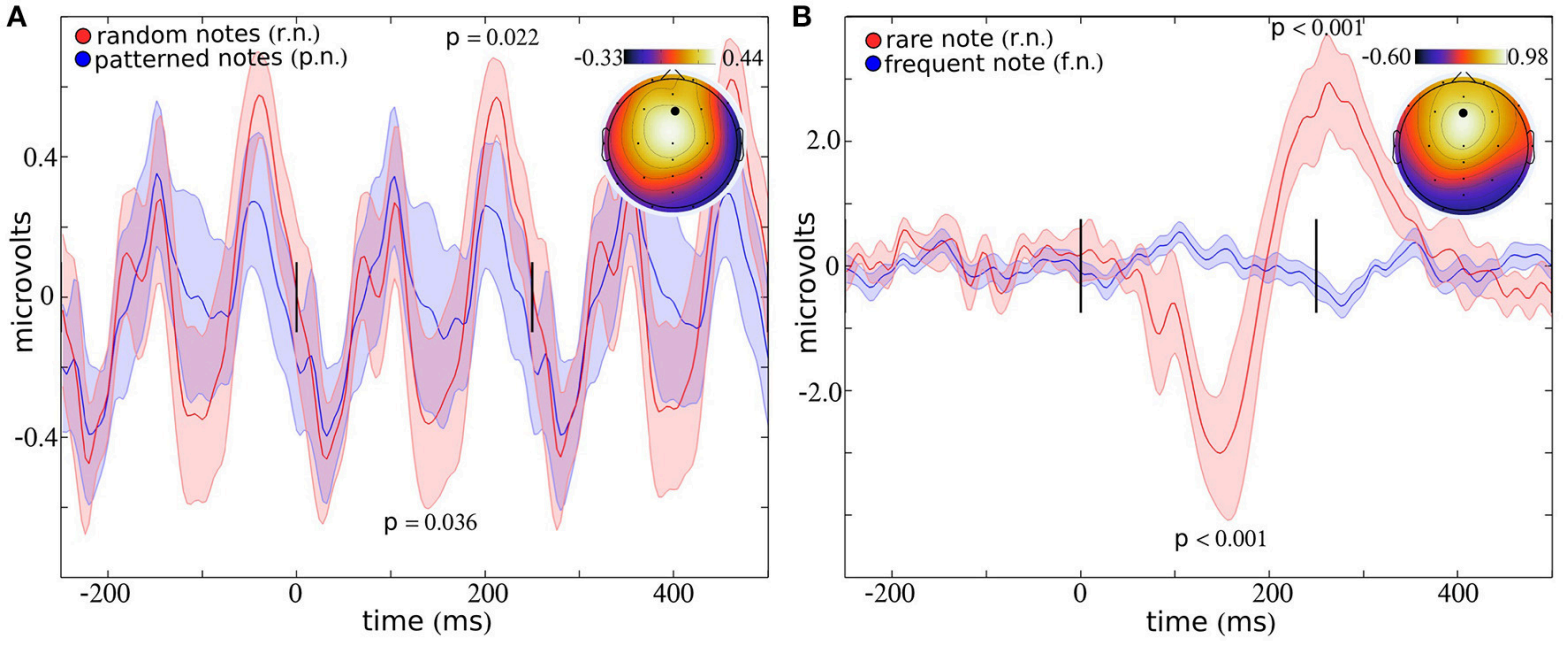
**ERP response to notes**. The ERP response to a sequence of notes presented with a musical pattern (blue) or a random pattern (red) is indicated in **(A)**, for electrode Fz (indicated by a black dot in the topographic plot). The ERP response to an infrequent note (red) presented within a series of frequent notes (blue) is indicated in **(B)**. Within each plot, the topography indicates the average amplitude around the full width half maximum (fwhm) at the peak. The lines at 0 and 250 ms indicate the onsets of the stimuli (presented at 4 Hz). Error bars represent the standard error.

Averaged across all musical and random notes (blocks 1–4), the largest peak response was observed over electrode Fz (Figure 2A), consistent with previous studies (Brattico et al., 2006; Virtala et al., 2014; Vuust et al., 2016). Four peaks appear within the interval from 0 to 250 ms, suggesting that the 4 Hz note sequence contributes to enhancement and/or phase locking of 8 Hz cortical oscillations (Figure 2A). This periodic structure is still present when the ERP is plotted using every other stimulus, which ensures that distinct stimuli were used to comprise the ERP responses that appear at −250, 0, and 250 ms (See Supplementary Figure 1A). Differences in ERP amplitudes for music and random notes appear to a greater extent as the delay between stimulus onset increases. For example, differences in ERP amplitudes failed to reach significance for peak 1 (at ~20 ms; *T* = 0.14; *p* = 0.896) and peak 2 (at ~100 ms; *T* = 0.25; *p* = 0.807). The negative peak at ~175 ms shows a 56.42% reduction for random notes compared to musical notes (*T* = 2.35; *p* = 0.036). This difference is less than the conventional uncorrected threshold of 0.05 but does not pass Holm–Bonferroni correction for multiple comparisons. The positive peak at ~200 ms statistically differs between music and random, with a 45.41% increase in response to random notes (*T* = 2.63; *p* = 0.022).

### ERP differences to rare and frequent notes

Averaged across all rare and frequent notes (block 5), the largest peak response was observed over electrode Fz (Figure 2B). Significant differences in ERP amplitudes are present for each of the two peaks which appear within the interval from 0 to 400 ms (Figure 2B). The peak response to the rare note is reduced 300.35% compared to the response to the frequent note (*T* = 5.44; *p* = 0.00015) at ~175 ms, and the response to the rare note increased 759.77% compared to the frequent note (*T* = 5.48; *p* = 0.00014) at ~300 ms.

### Relationship between sensitivity to music and sensitivity to rare stimuli

**Figure 3:**
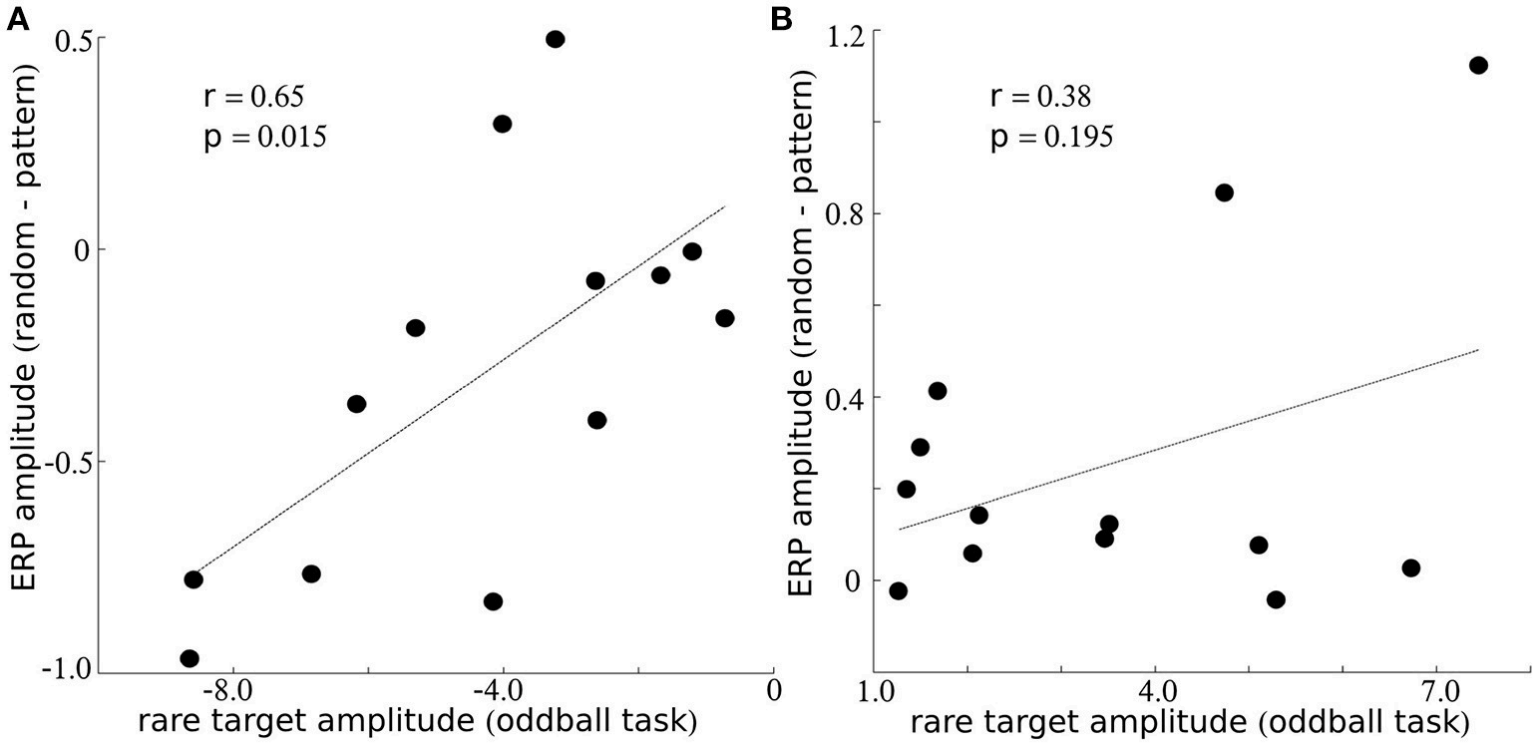
**Relationship between sensitivity to music and rare stimuli**. Within **(A,B)**, the y-axis indicates the amplitude difference between notes presented with a musical and random sequence and the x-axis indicates the amplitude to the rare stimulus during the oddball sequence. Results are presented for electrode Fz for the negative peak around ~175 ms within **(A)**, and for the positive peak around ~200 ms (music/random) or 300 ms (oddball sequence) within **(B)**.

The traditional oddball sequence (i.e., block 5) provides a broad measure of individuals cortical sensitivity to rare stimuli, while the musical and random sequence of notes provide a more nuanced measure of individuals sensitivity to subtle acoustic patterns. In order to explore relationships between these processes, we examined correlations between ERP amplitude differences in the music vs. random condition for the negative peak at ~175 ms (Figure 2A) and the negative peak observed for responses to the rare note (Figure 2B, in red). Statistical correlation was also computed between the amplitudes of the late positive peak that appeared at ~200 ms for blocks 1–4 and the amplitude of the response to rare stimuli at ~300 ms (block 5). Response amplitudes were significantly correlated for the negative peak at ~175 ms (*r* = 0.65; *p* = 0.015; Figure 3A), but did not reach statistical significance for the later positive peak (*r* = 0.38; *p* = 0.195; Figure 3B).

### Self-reported musical preference and experience

Three out of four self-report measures demonstrate a narrow distribution of responses across the 13 subject sample. Individuals generally reported that they liked music, with responses ranging from 4 (“indifferent”) to 7 (“I love it”). Individuals reported ability to detect the beat ranged from 3 (“sometimes”) to 5 (“always”), and only 4 out of 13 participants reported experience with a musical instrument. These three measures were excluded from the analysis due to the poor distribution of responses and limited sample. Individual's prior familiarity with scales spanned the full range of responses [i.e. from 1 (“not at all familiar”) to 7 (“very familiar”)]. Thus, we only focus on this self-report measure for further analysis.

### Relationship between sensitivity to music and familiarity with scales

Greater differences in amplitude between the pattern condition and random condition reflects a greater cortical sensitivity to features of the patterned sequence. We explored whether these responses were related to individual's behavioral experience with music by computing the correlation between their cortical sensitivity to music and individual's self-reported familiarity with musical scales. Correlations did not reach statistical significance for the peaks at ~175 ms (*r* = −0.45; *p* = 0.120) or ~200 ms (*r* = −0.11; *p* = 0.722; i.e., the two peaks which differed across conditions).

## Discussion

Within the present study, ERP responses were measured to individual guitar notes presented at 4 Hz with a musical pattern (i.e., sequences of notes comprising scales with repetition) or with a random pattern. The stimulus contained energy at 4 Hz and its harmonics (see Figure 1C), however ERP responses were primarily present at 8 Hz, with four peaks within the 250 ms interval corresponding to each individual note. The response at 8 Hz may have resulted due to entrainment of endogenous alpha oscillations to the periodic input. Statistically significant differences in the ERP response to individual notes were present for the positive peak at ~200 ms, with smaller absolute valued amplitudes within the pattern condition compared to the random condition.

These results are consistent with amplitude modulations of traditional ERP peaks [e.g., the mismatch negativity (MMN) and P300] measured within the auditory oddball sequence. Within this literature, the absolute value of ERP amplitudes increases for rare or unexpected stimuli compared to frequently presented stimuli (Polich, 2007; May and Tiitinen, 2010). Greater absolute valued amplitudes to random notes within the present study likely emerge due to the reduced ability to predict notes within the random sequence compared to the musical sequence. These findings suggest that greater processing resources were required to represent stimuli when they were presented randomly, and that individuals were better able to detect acoustic regularities to notes presented musically. Thus, the amplitude differences between the two conditions likely reflect individual's cortical sensitivity to guitar note melody. It is likely that similar effects would be observed using notes from other instruments, as long as they are organized the same way as the present study (i.e., within scales and with repetition).

We also measured ERP responses within the same subjects while they listened to a rare note embedded within a sequence of frequent notes. These findings allow us to determine whether the cortical sensitivity to complex musical structure overlaps with the sensitivity to simple irregularities reflected in the traditional auditory oddball sequence. ERP amplitudes to the rare stimulus topographically and temporally resembled well characterized ERP peaks [i.e., the mismatch negativity (MMN) and P300; Figure 2B]. Thus, the “steady-state” amplitude modulations to 4 Hz stimuli could be compared with ERP peaks which resemble those commonly labeled and reported in previous studies. Interestingly, ~175 ms amplitude differences (music minus random) were statistically correlated with MMN amplitudes, but amplitude differences to the positive peak at ~200 ms did not show a statistical relationship to the P300 response. Thus, the negative peak at ~175 ms appears sensitive to both complex musical patterns and simple statistical regularities. The positive peak at ~200 ms within the pattern condition appears sensitive to complex stimulus sequences while being unrelated to the P300 response that follows a rare stimulus embedded within a series of frequent stimuli. Thus, ERP response modulations to musical patterns partially overlap with the ERP peak modulations that reflect statistical regularities in the traditional “auditory oddball” sequence.

### Cortical entrainment to patterns

“Steady-state” responses could potentially emerge within all experiments within the present study since all stimuli were notes presented at 4 Hz. ERP responses to notes presented within the musical and random sequence appear to reflect this “steady-state” entrainment to the periodic input, with oscillations completing 2 periods within the interval that corresponds to 1 stimulus (250 ms). These oscillations may emerge due to phase alignment of 8 Hz alpha rhythms to each input. This oscillatory structure appears present to a lesser degree when the 4 Hz stimuli were presented with an oddball sequence (i.e., a rare note presented within a series of frequent/standard notes; compare Figure 2A with Figure 2B, and Supplementary Figure 1). While previous studies demonstrate the influence of input *frequency* on entrainment (Poeppel, 2003), the present findings suggest that entrainment is additionally influenced by stimulus *content*.

The less robust entrainment within the oddball sequence may be related to the repetitive nature of the stimulus sequence. The oddball sequence consists of a single note repeated continuously (90% of the time), with a rare note presented intermittently (10% of the time). Due to its repetitive nature, individuals may maintain a strong internal representation of the frequent note, which may improve their ability to suppress the note and/or reduce the need or utility of processing individual stimuli. These processes may contribute to the reduced response to the frequent stimulus observed within our study (Figure 2B) and within previous short ISI studies (Yabe et al., 1997; Wang et al., 2008).

In addition, more complicated stimulus sequences may require more advanced cognitive resources for processing. Previous studies indicate that stimuli are better detected when their onset aligns with the phase of EEG oscillations (Mathewson et al., 2009; Dugue et al., 2011; Neuling et al., 2012; Song et al., 2014), when neurons tuned to stimulus features are at their most excitable state (Lakatos et al., 2007; Haegens et al., 2011). Thus, cortical entrainment may facilitate or underlie the efficient processing of incoming stimuli, particularly when those stimuli are complex patterns.

### Potential advantages of shorter ISIs

Short ISI's are a prominent feature of SSEP studies, where different input frequencies can target brain networks with functionally distinct properties (Ding et al., 2006; Bridwell and Srinivasan, 2012). The 4 Hz presentation rate within the present study aligns well with speech prosody changes (0–4 Hz) and syllable boundaries (4–8 Hz), and the grouping of guitar notes into keys resembles the grouping of phonemes into words. Thus, the fast presentation frequency and musical complexity of the present paradigm may better reveal cortical processes specialized for speech and language (Yrttiaho et al., 2011).

The standard auditory oddball ERP response appears preserved despite the 4 Hz presentation rate of the present study, while the ERP response to guitar note sequences appears periodic (i.e., suggesting phase entrainment). Thus, the ERP response to the rare (i.e., oddball) stimulus extends temporally into the regions where the next stimulus appears (i.e., beyond 250 ms) indicating that cortical processes to the rare stimulus are preserved and prioritized at the expense of processes related to the subsequent frequent stimulus.

Generally, these findings indicate that ERP responses may be measured with a fast presentation rate, where a greater number of responses to rare stimuli may be measured within a fixed time interval. For example, 120 ERP responses to rare stimuli and 1,080 ERP responses to frequent stimuli were collected in 5 min within the present study. It is likely that the amplitude of these responses may be reduced with the faster presentation rate, as suggested by previous studies (Gonsalvez and Polich, 2002; Gomes et al., 2008; Rosburg et al., 2010; Pereira et al., 2014), but further studies may investigate whether these amplitude differences may be offset by reduced error estimates and enhanced statistical differences between the rare and frequent stimuli due to the larger number of responses collected.

### ERP's and self-reported musical experience and preference

The limited sample size and absence of a “musician” group may have limited our ability to identify relationships between ERP's and self-reported musical experience and preference. There appears to be a robust relationship between musicianship and cortical responses to music (Yu et al., 2015), with previous studies indicating more negative MMN amplitudes to pitch differences in violinists than non-violinists (Koelsch et al., 1999), and a more negative MMN to abstract changes in relative pitch within short melodies within musicians compared to non-musicians (Seppänen et al., 2007). Fujioka et al. (2004) reported that musicians MMN responses were more negative for changes in relative pitch within melodies, but did not statistically differ from non-musicians for pitch changes in a series of pure tones (Fujioka et al., 2004).

Generally, ERP response modulations may appear for both musicians and non-musicians for relatively simple musical features, but differences for more complicated musical patterns may only emerge when participants have a more extensive exposure to music (Brattico et al., 2009; Herholz et al., 2009; Hove et al., 2014; Vuust et al., 2016). The present findings indicate that ERP responses are modulated by guitar notes presented within musical scales with repetition, suggesting that non-musicians are sensitive to the musical features that are prominent within Western musical culture. Nevertheless, it is possible that musicians may have demonstrated a greater cortical sensitivity to these features compared to a sample of non-musicians.

## Conclusion

Individuals sensitivity to musical patterns were examined by comparing ERP responses to guitar notes presented at 4 Hz within a sequence with a musical pattern (i.e., within scales and with repetition) or within a random sequence. Cortical responses appeared to entrain to the periodic input, with two periods (i.e., four peaks) appearing within the 250 ms interval following each guitar note. The absolute value of ERP response magnitudes was reduced at ~200 ms for notes that appeared within the musical context. This amplitude difference between random and pattern conditions likely reflects individual's cortical sensitivity to guitar note melody. The negative peak at ~175 ms was statistically correlated with the MMN response, measured using a traditional oddball sequence, but the positive peak at ~200 ms did not statistically differ with P300 amplitudes measured within the same subject. Thus, the cortical sensitivity to musical patterns appears somewhat distinct from the cortical sensitivity to statistical regularities reflected in the traditional “auditory oddball” sequence.

## Author contributions

DB and DM were involved in conception and design of research; DB and DM analyzed data; DB and VC interpreted results of experiments; DB and DM prepared figures; DB drafted the manuscript; DB and VC edited and revised the manuscript; SP and VC supplied materials; DB, EL, DM, SP, and VC approved the final version of the manuscript; EL performed experiments.

### Conflict of interest statement

The authors declare that the research was conducted in the absence of any commercial or financial relationships that could be construed as a potential conflict of interest.
