# Supplementary material for: Cortical Sensitivity to Guitar Note Patterns: EEG Entrainment to Repetition and Key
Source: Front Hum Neurosci. 2017 Mar 1;11:90. doi: 10.3389/fnhum.2017.00090 (PMC5331856; doi:10.3389/fnhum.2017.00090)
Supplement: Supplementary file 4 [file Image1.PDF]

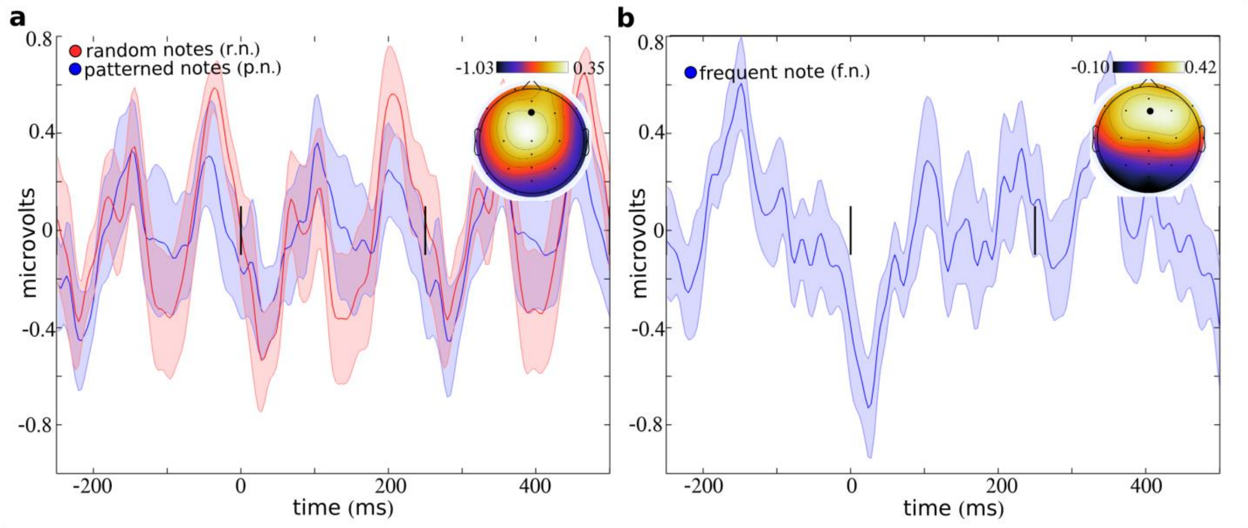

Supplementary Figure 1. ERP response to independent notes. The ERP response to a sequence of notes presented with a musical pattern (blue) or a random pattern (red) is indicated in a, for electrode Fz (indicated by a black dot in the topographic plot). ERP's were constructed from every other note, thus, the notes used to construct the ERP response at time 0 are different from the notes that contribute to the ERP response at time -250 ms and 250 ms. The presence of four peaks within the 250 ms interval of each stimulus further supports the notion of entrainment of ERP responses to these stimuli. ERP responses to every other standard stimulus within the oddball paradigm are presented within b, except when infrequent notes were presented in the note that precedes or follows the standard. These responses appear less consistent between neighboring notes, demonstrating reduced entrainment to these stimuli compared with the more complicated sequence of random and patterned notes in a. Within each plot, the topography indicates the average amplitude around the full width half maximum (fwhm) at the positive peak. The lines at 0 and 250 ms indicate the onsets of the stimuli (presented at 4 Hz). Error bars represent the standard error.
